# Current Advancements in Antitumor Properties and Mechanisms of Medicinal Components in Edible Mushrooms

**DOI:** 10.3390/nu14132622

**Published:** 2022-06-24

**Authors:** Jing Xu, Rui Shen, Zhuoya Jiao, Weidong Chen, Daiyin Peng, Lei Wang, Nianjun Yu, Can Peng, Biao Cai, Hang Song, Fengyuan Chen, Bin Liu

**Affiliations:** 1 School of Integrated Chinese and Western Medicine, Anhui University of Chinese Medicine, Hefei 230012, China; tojingxu@163.com (J.X.); shenrui28256@163.com (R.S.); zhuoya09136@163.com (Z.J.); caibiao@ahtcm.edu.cn (B.C.); 2 School of Pharmacy, Anhui University of Chinese Medicine, Hefei 230012, China; wdchen@ahtcm.edu.cn (W.C.); pengdy@ahtcm.edu.cn (D.P.); wanglei@ahtcm.edu.cn (L.W.); ynj2005288@sina.com (N.Y.); pengcan@ahtcm.edu.cn (C.P.); 3 Cancer Research Center, Beijing Chest Hospital, Capital Medical University/Beijing Tuberculosis and Thoracic Tumor Research Institute, Beijing 101149, China

**Keywords:** edible and medicinal fungi, malignant tumors, anticancer, molecular mechanism, clinical application

## Abstract

Edible and medicinal fungi, a group of eukaryotic organisms with numerous varieties, including *Coriolus versicolor*, *Ganoderma lucidum*, *Cordyceps sinensis*, *Pleurotus ostreatus*, and *Grifola frondosa*, have been demonstrated to possess a board range of pharmaceutical properties, including anti-virus, anti-inflammation, and neuroprotection. Moreover, edible and medicinal fungi have been traditionally consumed as food to provide multiple nutrients and as drugs owing to having the activities of invigorating blood circulation, reinforcing the healthy qi, clearing away heat, and eliminating stasis for thousands of years in China. Malignant tumors, well-known as the second leading cause of death globally, accounted for nearly 10 million deaths in 2020. Thus, in-depth exploration of strategies to prevent and treat cancer is extremely urgent. A variety of studies have reported that the main bioactive components of edible and medicinal fungi, mainly polysaccharides and triterpenoids, exhibit diverse anticancer activities via multiple mechanisms, including inhibition of cell proliferation and metastasis, induction of apoptosis and autophagy, reversing multidrug resistance, and regulation of immune responses, thus suggesting their substantial potential in the prevention and treatment of cancer. Our review summarizes the research progress on the anticancer properties of edible and medicinal fungi and the underlying molecular mechanism, which may offer a better understanding of this field. Additionally, few studies have reported the safety and efficacy of extracts from edible and medicinal fungi, which may limit their clinical application. In summary, there is a need to continue to explore the use of those extracts and to further validate their safety and efficacy.

## 1. Introduction

### 1.1. Medicinal Effects of Edible and Medicinal Fungi

Fungi, a group of eukaryotic organisms with numerous varieties, including microorganisms and the more familiar mushrooms, not only play a vital role in balancing the environment but also generate great economic benefits as they can be consumed as food and generate antibiotics and other pharmaceutical products. In summary, fungi are of great environmental and medical importance, which deserve deeper exploration. In particular, edible and medicinal fungi, a type of healthy food, are not only edible with abundant nutrients, including protein, vitamins, minerals, and fat, but also display exciting medicinal effects due to containing multiple bioactive substances, such as polysaccharides and immunoregulatory proteins [1,2]. For example, *Ganoderma lucidum,* also called “Lingzi” in China, has been reported to exert diverse therapeutic properties in multiple diseases [3], and *Poria cocos,* also named as “Fuling” in Chinese, can induce diuresis to remove edema [4]. *Grifola frondosa*, widely known as hen of the woods in Chinese and maitake in Japanese, has been documented to possess diverse pharmacological effects, including anti-tumor, anti-inflammatory, anti-allergic, and immune-boosting effects [5].

### 1.2. The Role of Edible and Medicinal Fungi in Traditional Uses

Importantly, a wide range of edible and medicinal fungi, including *Coriolus versicolor*, *Ganoderma lucidum*, *Cordyceps sinensis*, *Pleurotus ostreatus*, *Grifola frondosa*, *Ganoderma applanatum*, *Lentinus edodes*, *Auricularia auricula*, *Flammulina velutipe*, and so on, has a long history of medicinal use in China and other Asian countries [6]. According to volume 194 of Puji Fang, a famous medical prescription dating from 14th century, a combined administration of *Poria cocos* with *Polyporus umbellatus* has been used to treat malignant tumors originated from abdomen [7]. Consistent with previous records, volume 8 of Variorum of Shennongs Classic of Materia Medica recorded the anti-tumor effects of *Poria cocos* in treating breast cancer [8]. Additionally, owing to having the activities of invigorating blood circulation, *Phellinus linteus* has been utilized to treat cancer originating from the female reproductive system according to volume 28 and 36 of Compendium of Materia Medica [9]. In addition, volume 221 of Puji Fang recorded the pharmaceutical benefits of *Ganoderma lucidum* in treating advanced cancer by reinforcing the healthy qi and promoting blood circulation [7]. Moreover, in volume 49 of Jingyue’s Complete Works, tabasheer has been used to treat lung cancer due to its pharmaceutical properties, namely clearing away heat and eliminating stasis [10].

Currently, recent advancements in molecular biology and chemical analysis contribute to the in-depth understanding of the effects and the underlying mechanisms of TCM. For instance, according to previous studies, many edible and medicinal fungi contain a variety of bioactive components, including polysaccharides, phenols, purine, and immunoregulatory proteins, thus exerting diverse pharmacological activities [11]. It is worth mentioning that the anti-tumor effects of edible and medicinal fungi have been confirmed in both in vitro and in vivo models of lung cancer [12], liver cancer [13], and breast cancer [14]. Moreover, fungal polysaccharides, the major bioactive ingredients in many types of edible and medicinal fungi, can display anti-tumor effects by improving the immunity of the body [15,16,17].

Although significant improvement has been made in developing effective anticancer strategies, the prognosis of tumor-bearing patients still remains unsatisfactory due to high mortality rate. Furthermore, patients who undergo anticancer therapies may experience a variety of side effects, including pain, fatigue, loss of weight, and so on. Considering that edible and medicinal fungi have been utilized safely for thousands of years in treating various diseases including cancer, our article summarizes research progress of the anticancer activities of edible and medicinal fungi and the potential mechanisms in hope of providing insight for further prevention and treatment of cancer.

## 2. Anticancer Activities of Edible and Medicinal Fungi

Cancer, a group of diseases characterized by uncontrolled cell growth with a tendency to invade adjoining parts of the body, arises from the conversion of normal cells into cancer cells. Although the exact reason why cancer happens is not fully illustrated, current studies have suggested that the incidence of cancer may be related to aging, chemicals, radiation exposure, infections, unhealthy diet, family history, being overweight, and so on. Currently, considering the type, location, stage, and size of the malignant tumor, cancer treatments include surgery, chemotherapy, radiotherapy, hormone therapy, biological therapy, or polytherapy. Cancer surgery, as the oldest form of cancer treatment, aims to remove cancer or possible nearby tissue, which is fairly effective in eliminating cancer cells before spreading to lymph nodes or distant sites. Moreover, surgery is usually followed by radiotherapy or chemotherapy to ensure all malignant cells have been removed [18]. Radiotherapy is a therapy that uses concentrated radiation beams to destroy or kill cancer cell, thus eliminating or shrinking tumors. For instance, given that nasopharyngeal carcinoma (NPC) is obviously sensitive to radiotherapy, radiotherapy is an effective therapeutic strategy in treating early-stage NPC [19,20]. Chemotherapy, one of the major types for cancer treatment, which uses one or multiple powerful chemicals to destroy fast-growing cancer cells, is often given as an adjuvant treatment following surgery or radiation [21]. It is worth mentioning that both radiotherapy and chemotherapy can cause side effects, including vomiting, nausea, cognitive dysfunction, hair loss, and cognitive changes. Biological therapy, a rapidly emerging area of cancer research, functions to induce the immune system to attack or recognize cancer cells, which includes targeted drug therapy, gene therapy, chimeric antigen receptor (CAR) T-cell therapy, and cancer vaccines [22,23]. Endocrine therapy, also called hormone therapy, is a treatment that refers to the administration of medicines to lower or block the amounts of hormones to treat breast or prostate cancer [24]. Although the development of diagnostic technologies and therapeutic strategies has improved the survival of patients experiencing primary malignant tumors, the toxic side effects of anticancer therapies and unsatisfactory outcomes in advanced-stage cancer patients have motivated notable efforts in exploring novel cancer therapies. To date, traditional Chinese medicine, a system of medicine that is characterized as a personalized and precision medicine, has been reported to extend life expectancy and improve quality of life.

Interestingly, recently studies have suggested that polysaccharides, bioactive ingredients extracted from medicine and edible fungi, can exert anticancer activities via activation of T lymphocytes, macrophages, and NK cells. Niu et al. suggested that polysaccharides isolated from *Bletilla ochracea Schltr* obviously suppressed tumor xenograft growth in vivo by promoting CD4+ T cell proliferation in the spleen of the tumor-bearing mice, thus indicating that *Bletilla ochracea Schltr* could be a potential immunomodulatory agent in cancer treatment [25]. In addition, a growing number of reports have indicated that triterpenoids, a class of natural compounds with a carbon skeleton based on six isoprene units which could be found in medicine and edible fungi, could exhibit anti-tumor effects against many cancer cells via induction of apoptosis and cell cycle arrest, suppression of metastasis, and so on. For example, *Ganoderma lucidum triterpenoids* have a broad spectrum of inhibitory effects against a variety of cancers, including oral mucosal cancer [26], prostate cancer cells [27], and liver cancer [28]. Concordantly, triterpenes isolated from *Poria cocos*, a widely used medicinal fungus in TCM owing to its diverse pharmaceutical activities, has been demonstrated to suppress the progression of pancreatic cancer [29], breast cancer [30], and lung cancer [31], thus suggesting it could be considered as a novel therapeutic agent in cancer treatment (Table 1).

## 3. The Mechanism of Edible and Medicinal Fungi in Inhibiting Cancer Progression

### 3.1. Inhibition of Cell Proliferation

Given that the common defining feature of cancer cells is uncontrolled cell growth, many kinds of anticancer drugs are designed to inhibit the relentless proliferation of cancer cells. Collectively, recent studies have indicated that a variety of extracts or compounds from edible and medicinal fungi can prevent or stop the proliferation of many different cancer cells.

*Auricularia auricula-judae*, also named as Huaier in Chinese, has served as a complementary therapeutic agent for liver cancer in recent decades. Shan et al. uncovered that Huaier suppressed cellular proliferation of hepatocellular carcinoma (HCC) cells, including Bel-7404, Bel-7402, and SMMC-7721 cells, promoted cell apoptosis of HCC cells, and inhibited HCC cells migration. Further mechanism study revealed Huaier treatment promoted the translocation of yes-associated protein 1 (YAP1) from nucleus to cytoplasm and facilitated Lats1/2-mediated YAP1 phosphorylation, thus ultimately resulting in a reduced level of YAP1 [45]. Additionally, *Auricularia auricula*, a medicinal and edible homologous fungus, contains various bioactive agents, including terpenes, polysaccharides, adenosine, sterols, and so on. A very recently published study documented that the anticancer properties of *A. auricula* were associated with downregulation of JUN, TLR4, and MYD88 expression [46]. FIP-fve, an immunomodulatory protein extracted from *Flammulina velutipes*, which is one of the most-produced fungi globally, has been demonstrated to exhibit immunomodulatory properties via induction of chemokine and cytokine production. Chang et al. recently suggested that FIP-fve suppressed lung cancer progression. In-depth investigation found that the anti-tumor activity of FIP-fve was achieved via p53 mediated suppression of lung cancer cells A549 proliferation and RacGAP1 mediated inhibition of A549 cell migration [47]. More interestingly, three polysaccharides obtained from *Flammulina velutipes* can obviously promote cell proliferation and stimulate the phagocytic activity of macrophage RAW 264.7 cells [48].

### 3.2. Induction of Apoptosis and Autophagy

Autophagy refers to a self-degradative process that sequesters organelles, lipids, and proteins in autophagosome for degradation, thus contributing to maintain cellular homeostasis under nutrient stress [49]. Increasing studies have suggested that autophagy plays a paradoxical role in tumorigenesis, acting as both a tumor-promoter and a tumor-suppressor, dependent on the stage of cancer progression and the cellular context [50]. At the early stage of tumorigenesis, autophagy functions as a tumor suppressor owing to degradation of damaged cellular parts and proteins. However, at the advanced stage, autophagy facilitates tumorigenesis by ameliorating stress in the tumor microenvironment. *Ganoderma lucidum polysaccharide* (GLP), well-known as one of the major bioactive ingredients isolated from *G. lucidum*, has been reported to induce autophagy initiation and suppress autophagic degradation in colorectal cancer (CRC) cells. Moreover, a deep investigation suggested that reduced autophagosome–lysosome fusion was responsible for GLP-induced autophagy, which was mediated via activation of MAPK/ERK pathway, thus indicating GLP could be a promising adjuvant or direct autophagy inhibitor in CRC treatment [35].

**Figure 1:**
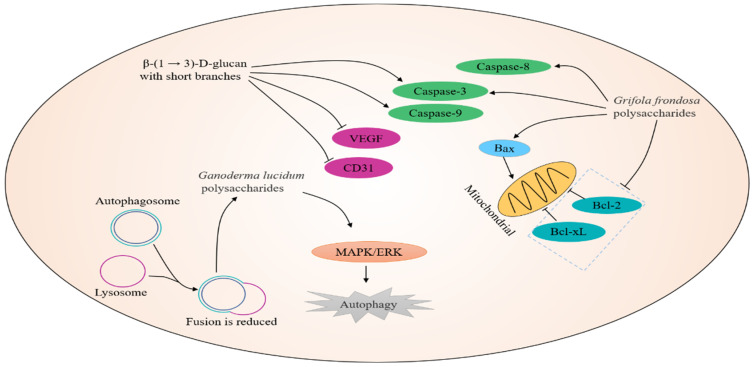
Mechanisms in terms of apoptosis and autophagy induction by the active ingredients of edible and medicinal fungi. Reduced fusion of autophagosomes and lysosomes leads to the induction of autophagy by *Ganoderma lucidum* polysaccharides through MAPK/ERK pathway. *Grifola frondosa* polysaccharides-incubated breast cancer cells exhibit an increased level of Bax, caspase-3, caspase-8, and a decreased expression of Bcl-2 and Bcl-xL. β-(1→3)-D-glucan with short branches inhibits the growth and induces the apoptosis of HCC cell line H22 by increasing caspase-3/9 levels and decreasing VEGF and CD31 levels.

Apoptosis is a form of orchestrated and programmed cell death that eventually results in the elimination of cells, which can occur both in physiological and pathological circumstances. It is universally acknowledged that too little apoptosis represents a substantial causative factor for cancer development [51]. Consequently, apoptosis could be an essential strategy for anti-tumor treatments [52]. Moreover, the initiation of apoptosis can be divided into two major pathways, namely the extrinsic (death receptor-mediated) and the intrinsic (mitochondria-dependent) pathways. The Bcl-2 family, characterized by sharing Bcl-2 homology (BH) domains, are the central gatekeepers of mitochondria-dependent apoptosis, which include Bcl-2 (apoptotic suppressor gene) and Bax (pro-apoptotic gene). *Grifola frondosa* polysaccharides (GFPs)-treated breast cancer cells, including MCF-7 and MDA-MB-231 cells, exhibited a reduced cell growth, an increased apoptosis, and reactive oxygen species (ROS) accumulation. Identical to cells’ phenotype, GFPs incubation resulted in an increased level of Bax, cleaved caspase-3, caspase-8, and a decreased expression of Bcl-2 and Bcl-xL. Moreover, the anticancer activities of GFPs were confirmed in vivo as GFPs inhibited the growth of MCF-7 tumor xenografts in nude mice. Taken together, GFPs could be a potential therapy strategy for breast cancer [53]. The water soluble *β*-(1→3)-D-glucan with short branches (AF1) obtained from *Auricularia auricula* inhibited the growth of HCC cell line H22 and induced H22 apoptosis. Further investigations suggested that AF1-incubated H22 cells displayed an increased level of caspase-3/9 and a reduced level of vascular endothelial growth factor (VEGF) and cluster of differentiation 31(CD31) [54]. Interestingly, Kang et al. documented that the tumor suppressive activities of *Auricularia auricula* were mediated by peroxiredoxin1-induced apoptosis [55]. Importantly, a clinical trial reported that polysaccharides isolated from Huaier obviously improved survival, treatment response rate, and immunity of patients with gastrointestinal cancers (GICs), thus indicating that Huaier could be considered as a good adjuvant [56]. Wang et al. revealed that cordyceps acid, one of the main bioactive ingredients of *Cordyceps sinensis*, induced apoptosis of human lung cancer A549 cells and suppressed growth of A549 cells transplanted in nude mice via regulation of Nrf-2/HO-1/NLRP3/NF-κB pathway [57]. Besides, *Cordyceps sinensis* polysaccharide (CSP) promotes apoptosis and autophagy flux blockage of human colon cancer HCT116 cells, thus indicating CSP could be a promising therapeutic component for the treatment of colon cancer [58] (Figure 1).

### 3.3. Inhibition of Metastasis

Cancer metastasis, a process that cancer cells spread from the primary lesion to other parts of the body, is responsible for one of the main causes of cancer death, which is a dynamic and stepwise process that involves a complex interplay between cancer cells and the tumor microenvironments [59]. It is worth mentioning that some extracts from edible and medicinal fungi could exert inhibitory effects on cancer metastasis depending on the cell contents.

Luo et al. reported that the anti-metastasis activities of aqueous extract from *Coriolus versicolor* (CV) as well as its protective functions in breast cancer-induced bone destruction. Moreover, an increased level of interleukin (IL)-2, 6, 12, interferon-γ (IFN-γ), and tumor necrosis factor-α (TNF-α) in the spleen lymphocytes of CV-treated tumor-bearing mice reflected immunomodulation properties of CV aqueous extract [60]. Additionally, polysaccharide-rich extracts isolated from CV and *Grifola frondosa* (GF) have been reported to inhibit human colon cell proliferation, migration, and invasion, and anti-metastasis activities of above extracts may be driven by increasing the expression of E-cadherin [61]. Notably, the migration and invasiveness of gastric cancer (GC) cells were suppressed by aqueous Huaier extract. Furthermore, aqueous Huaier extract partly reversed epithelial-mesenchymal transition (EMT) by suppressing Twist, as confirmed by an elevated level of E-cadherin (an epithelial marker) and a reduced expression of N-cadherin and vimentin (mesenchymal markers) [62]. Interestingly, the anticancer activities of selenium-enriched polysaccharide fraction produced by *Pleurotus ostreatus* were also mediated via inhibition of EMT transition [63]. Furthermore, Cai et al. demonstrated that water extracts of *Cordyceps sinensis* (WECS) have anti-metastasis properties, as characterized by reduced metastatic tumor nodules and increased survival rate of 4T1 tumor-bearing mice. In addition, in-depth studies suggested that reduced metastasis-related cytokines may contribute to the anticancer effects of WECS [64].

### 3.4. Regulation of the Immune System

**Figure 2:**
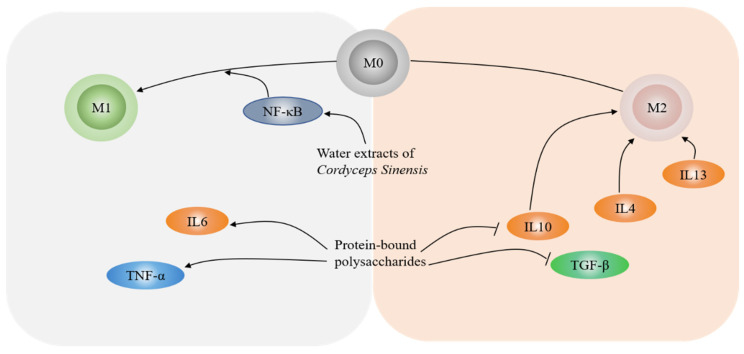
Antitumor mechanisms associated with regulation of macrophage polarization. The levels of IL-6 and TNF-α were elevated and the expression of IL-10 and TGF-β was decreased in protein-bound polysaccharides (PBP) cultured breast cancer cells. PBP regulates M1/M2 polarization and exhibits antitumor activities during breast cancer progression. Water extracts of *Cordyceps sinensis* exert anti-breast cancer effects by regulating the differentiation of macrophages to M1-like macrophages through modulation of NF-κB signaling pathway.

The human immune system has the ability to recognize and attack cancer cells, but cancer cells can acquire the capability to evade immune surveillance. Therefore, cancer immunotherapy, a form of cancer therapy that aims to boost the immune system to eliminate cancer cells, has gradually become a non-negligible part of cancer therapies [65]. It is noteworthy that a wide range of edible and medicinal fungi have been documented to exhibit anti-cancer activities via regulation of immune responses. Polysaccharopeptide (PSP) extracted from *Coriolus versicolor* can induce predominantly pro-inflammatory cytokines by promoting cytotoxic activities of natural killer and CD8+ T cells [66]. A variety of studies suggested that macrophages play a vital role in tumor microenvironment (TME). Generally, M1-polarized macrophages exert anti-tumor effects through eliminating and destroying phagocytosed tumor cells, while M2-like macrophages, activated by IL-4, IL-10 and IL-13, resemble tumor-associated macrophages (TAMs), which promote tumor progression though immunosuppressive functions. Recently, Jędrzejewski et al. reported that protein-bound polysaccharides (PBP) from *Coriolus versicolor* can govern M1/M2 polarization, thus exhibiting anti-tumor activities in breast cancer progression, as revealed by an elevated level of IL-6, TNF-α (pro-inflammatory markers), and a decreased expression of IL-10, transforming growth factor-β (TGF-β), and arginase 1 (M2 markers) after PBP treatment [67]. Accordingly, the tumor suppressive role of WECS in breast cancer progression was achieved by governing the differentiation of macrophages to M1-like macrophages via regulation of NF-κB signaling pathway [68] (Figure 2). Additionally, NK-cell cytotoxicity induced by *Pleurotus ostreatus* glucan was mediated by activation and induction of IFNγ and NO [69]. Some of the active ingredients in edible and medicinal mushrooms may play a role as biological response modifiers in cancer treatment. D-fraction is a polysaccharide obtained from *Grifola frondosa*, which can activate macrophages, NK, and other cells to enhance the immune system and inhibit tumor activities [70,71]. At the same time, it can be used in combination with mitomycinc and cisplatin to enhance the anticancer effects of drugs [72,73].

### 3.5. Reversal of Multidrug Resistance and Increasing Sensitivity to Chemotherapy

Multidrug resistance (MDR), a refractory outcome of long-term chemotherapy, refers to a phenomenon by which cancer cells exhibit resistance to multiple chemotherapeutic agents, thus ultimately leading to recurrence of malignant tumors [74]. Although the mechanisms underlying MDR are far from clear, accumulating studies have reported that DNA damage repair, genetic factors, enhanced drug efflux, and reduced apoptosis are responsible for the development of MDR. As a result, formulating novel strategies to overcome MDR constitutes a crucial aim of anticancer research [75,76]. ATP-binding cassette (ABC) transporters, a group of transmembrane proteins that are responsible for the transport of substances across the cell membrane, have been reported to be closely associated with MDR by both clinical and experimental studies. Consequently, targeting ABC transporters can be a potential strategy to reverse MDR, thus eventually contributing to cancer treatment [77]. Notably, a variety of edible and medicinal fungi have been reported to negatively modulate some crucial factors associated with MDR. Ganoderma acid B extracted from *Ganoderma lucidum* can reverse multidrug resistance of HepG2/ADM cells to doxorubicin by inhibiting the transport function of ABCB1, a type of ABC transport protein [78]. Sadava et al. have reported extracts of several species of *Ganoderma* suppressed the growth of both drug-sensitive and drug-resistant small-cell lung cancer (SCLC) cells, thus indicating that *Ganoderma* could be a promising agent to reverse MDR [79]. In addition, co-administration with cordycepin, a bioactive compound in *Cordyceps* obviously promoted gemcitabine/5-FU-induced human gallbladder cancer sensitivity [80]. Chiu et al. reported that a fungal protein from *Ganoderma microsporum* suppressed the growth of MDR subline by promoting Akt/mTOR signaling pathway mediated apoptosis and autophagy [81]. Moreover, incubation of drug-resistant KBV200 cells with eight triterpenoids from *Poria cocos* induced vincristine-induced apoptosis [82]. Interestingly, a combined administration of pachymic acid and dehydrotumulosic acid purified from *Poria cocos* with doxorubicin (DOX) can enhance the sensitivity of drug-resistant MCF cells via inhibition of P-glycoprotein (P-gp)-mediated MDR [83] (Table 2).

## 4. Clinical Research

Edible and medicinal fungi, a group of organisms that are appraised for their medicinal and economic benefits, have attracted considerable attention across researchers. Moreover, owing to having the properties of antitumor, anti-oxidation, and anti-inflammation, many clinical trials have been designed to assess the efficacy and safety of extracts from edible and medicinal fungi. In clinical practice, Papilocare, a *Coriolus versicolor*-based vaginal gel, has shown good efficacy and high safety in overall HPV clearance by a multicenter, randomized, and controlled trial [84]. Furthermore, a clinical study documented the immunological benefits of Ganoderma spore powder in post-operative patients with breast and lung cancer, as characterized by a lower expression level of COX2 and TGF-β1 (immunosuppressive factors) and high AGR/NLR ratio (a favorable prognostic indicator) [85]. Additionally, a phase II clinical trial performed by Liu et al. suggested a preliminary safety of Reishi and Privet Formula (RPF) in maintaining the quality life of patients with non-small-cell lung carcinoma (NSCLC) undergoing chemotherapy [86]. Further, a phase I trial of white button mushroom (WBM) in 36 patients with recurrent prostate cancer revealed that WBM suppressed prostate-specific antigen (PSA) as well as potentially modulated the biology of some prostate cancer patients [87]. Interestingly, 47 CRC patients were administered with *G. lucidum* at a dose of 5.4 g/day for 12 consecutive weeks. In addition, the studies documented that oral intake of *G. lucidum* extracts increased the expression of IL-2, IL-6 and IFN-γ but decreased the level of IL-1 and TNF-a, indicating that *G. lucidum* could be a promising immuno-modulating agent [88]. Moreover, advanced HCC patients were administered a daily dose of 2.4 g *Coriolus versicolor* for 12.1 weeks. Although there was no significant difference in time to median disease progression (TTP) between 2 groups, patients with CV administration generally exhibited a better quality of life [89].

Despite a growing number of studies reporting the biosynthesis, purification, and pharmaceutical effects of extracts isolated from edible and medicinal fungi, there are limited pharmacokinetic studies of these extracts. A recent study reported that ganoderic acid (GA), one of major bioactive ingredients in *Ganoderma lucidum*, could be absorbed and distributed extensively in a variety of tissues after oral administration. For instance, a study which aimed to assess the pharmacokinetics of ganoderic acids A and F suggested the *T_max_* of both ganoderic acids was nearly 0.5 h and *t_1/2_* was 37 min and 28 min, respectively [90]. Polysaccharide K (PSK), extracted from *Coriolus versicolor*, is commonly utilized as an adjuvant immunotherapy in cancer treatments. Moreover, pharmacokinetic explorations revealed that PSK was mainly digested by gastrointestinal (GI) tract and distributed extensively in different tissues, including bone, liver, brain, and so on. Breakdown products with low molecular weight could be found in the blood 2 h after intake and larger products were found after 4 h [91]. In addition, a study performed by Wang et al. reported main pharmacokinetic parameters of pachymic acid, a bioactive triterpenoid isolated from *Poria cocos*, after oral intake. The half-life was 4.96 ± 1.33 h and *t_max_* was 0.75 ± 0.14 h [92].

## 5. Conclusions and Prospects

Given that a variety of edible and medicinal fungi can exert broad-spectrum anticancer properties, some edible and medicinal fungi have been consumed in alternative medicine in cancer treatment. Nevertheless, there is still much scope for experimental and clinical research for wide application of edible and medicinal fungi.

Previous studies have reported edible and medicinal fungi contain various bioactive ingredients, which can exhibit antitumor effects through multiple mechanisms, including induction of apoptosis, inhibition of cancer metastasis and invasion, reversal of multidrug resistance, and regulation of immune responses. Consequently, extensive studies are needed to clarify the specific mechanism of antitumor actions of different active components from edible and medicinal fungi. Additionally, owing to low concentrations of bioactive ingredients and complicated extraction procedures, methods for the enrichment of bioactive molecules need to be further improved. Currently, only a small portion of edible and medicinal fungi has been studied, thereby, more research needs to be carried out to explore the remaining fungi. Last but not least, we need to scale up the clinical trials of food-drug fungi to further evaluate their safety and efficacy, in hope of providing references and hope to cancer prevention and treatment.

## 6. Highlights

- Edible and medicinal fungi have been shown to possess a variety of medicinal properties for millennia.
- The main active components of edible and medicinal fungi, namely, polysaccharides and triterpenes, exhibit a variety of antitumor activities through multiple mechanisms, including inhibition of cell proliferation and metastasis, induction of apoptosis and autophagy, reversing multidrug resistance and regulation of immune responses.
- In-depth exploration of the anticancer properties and molecular mechanisms of edible and medicinal fungi can be beneficial for cancer prevention and treatment.

## Figures and Tables

**Table 1:** Summary of the anticancer activities of edible and medicinal fungi.

Name	Main Active Components	Cancer	Target/Mechanism	Ref.
*Coriolus versicolor*	Polysaccharide	ER-positive breast cancer and amelanotic melanoma cells	Induce RIPK1/RIPK3/MLKL-mediated necroptosis	[32]
*Coriolus versicolor*	Polysaccharide	Amelanotic melanoma cells	Trigger caspase-independent cell death pathway	[33]
*Coriolus versicolor*	Polysaccharide	Tumor cell lines	Induce cell cycle arrest and apoptosis	[34]
*Ganoderma lucidum*	Polysaccharide	Colorectal cancer	Activate MAPK/ERK signaling pathway, induce autophagosome accumulation and apoptosis	[35]
*Ganoderma lucidum*	Polysaccharide	Breast cancer	Combination with paclitaxel inhibits tumor metabolism through intestinal microbiota remodeling	[36]
*Ganoderma lucidum*	Polysaccharide	Lung cancer	Induce degradation of TGFβ and EGF receptors via proteasome and lysosome	[37]
*Ganoderma lucidum*	Triterpene	Prostate cancer	Regulate matrix metalloproteinases	[27]
*Ganoderma lucidum*	Triterpene	Colorectal cancer	Induce apoptosis	[38]
*Ganoderma lucidum*	Triterpene	Lung cancer	Attenuate tumor angiogenesis	[39]
*Ganoderma applanatum*	Polysaccharide	Breast cancer	Induce cell apoptosis through intrinsic mitochondrial apoptosis and MAPK signaling pathway.	[40]
*Poria cocos*	Polysaccharide	Leukemic cells	Inhibit growth and induce differentiation through increasing IFN-γ and TNF-α cytokines	[41]
*Poria cocos*	Polysaccharide	Liver cancer	Decrease ALB protein expression and increase VEGFA level	[42]
*Poria cocos*	Polysaccharide	Breast cancer	Inhibit invasion and migration	[43]
*Poria cocos*	Triterpene	Lung cancer	Induce cell apoptosis	[31]
*Poria cocos*	Triterpene	Pancreatic cancer	Decrease the expression of KRAS and matrix metalloproteinase-7	[29]
*Poria cocos*	Triterpene	Prostate cancer	Induction of apoptosis	[44]

**Table 2:** Summary of extracts from edible and medicinal fungi in combination with chemotherapeutic drugs.

Drugs	Cancer	Extracts	Mechanisms	Ref.
Doxorubicin, vincristine, paclitaxel.	Liver cancer	Ganoderenic acid B	Inhibition of the transport function of ABCB1	[78]
Doxorubicin	Breast cancer	Ganoderenic acid B	Inhibition of the transport function of ABCB1	[78]
Gemcitabine,5-fluorouracil	Gallbladder cancer	Cordycepin	AMPK activation and MDR degradation	[80]
Docetaxel	Non-small cell lung cancer	A fungal protein from *Ganoderma microsporum*	Inhibition of Akt/mTOR signaling pathway	[81]
Vincristine	Human oral epidermoid carcinoma	Triterpenoids	Inhibition of the P-gp function	[82]
Doxorubicin	Breast cancer	Pachymic acid and dehydrotumulosic acid	Inhibition of the P-gp function	[83]

## Data Availability

Not applicable.

## Abbreviations

NPC, nasopharyngeal carcinoma; CAR, chimeric antigen receptor; TCM, traditional Chinese medicine; HCC, hepatocellular carcinoma; YAP1, yes-associated protein 1; GLP, *Ganoderma lucidum* polysaccharide; CRC, colorectal cancer; BH, Bcl-2 homology; ROS, reactive oxygen species; GFPs, *Grifola frondosa* polysaccharides; GICs, gastrointestinal cancers; CSP, *Cordyceps sinensis* polysaccharide; CV, *Coriolus versicolor*; GF, *Grifola frondosa*; GC, gastric cancer; EMT, epithelial-mesenchymal transition; WECS, water extracts of *Cordyceps sinensis*; PSP, Polysaccharopeptide; TAMs, tumor-associated macrophages; MDR, multidrug resistance; ABC, ATP-binding cassette; SCLC, small-cell lung cancer; DOX, doxorubicin; WBM, white button mushroom; PSA, prostate-specific antigen; transforming growth factor-β, TGF-β; natural killer cells, NK cells; interferon-γ, IFN-γ; tumor necrosis factor-α, TNF-α; interleukin-2, IL-2; vascular endothelial growth factor, VEGF; cluster of differentiation 31, CD31.
